# Avian influenza A (H9N2): computational molecular analysis and phylogenetic characterization of viral surface proteins isolated between 1997 and 2009 from the human population

**DOI:** 10.1186/1743-422X-7-319

**Published:** 2010-11-15

**Authors:** Azeem M Butt, Samerene Siddique, Muhammad Idrees, Yigang Tong

**Affiliations:** 1 National Centre of Excellence in Molecular Biology (CEMB), University of the Punjab. 87 - West Canal Bank Road, Thokar Niaz Baig, Lahore, 53700, Pakistan; 2 State Key Laboratory of Pathogen and Biosecurity, Beijing Institute of Microbiology and Epidemiology, Beijing, 100071, PR China

## Abstract

**Background:**

H9N2 avian influenza A viruses have become panzootic in Eurasia over the last decade and have caused several human infections in Asia since 1998. To study their evolution and zoonotic potential, we conducted an *in silico *analysis of H9N2 viruses that have infected humans between 1997 and 2009 and identified potential novel reassortments.

**Results:**

A total of 22 hemagglutinin (HA) and neuraminidase (NA) nucleotide and deduced amino acid sequences were retrieved from the NCBI flu database. It was identified that mature peptide sequences of HA genes isolated from humans in 2009 had glutamine at position 226 (H3) of the receptor binding site, indicating a preference to bind to the human α (2-6) sialic acid receptors, which is different from previously isolated viruses and studies where the presence of leucine at the same position contributes to preference for human receptors and presence of glutamine towards avian receptors. Similarly, strains isolated in 2009 possessed new motif R-S-N-R in spite of typical R-S-S-R at the cleavage site of HA, which isn't reported before for H9N2 cases in humans. Other changes involved loss, addition, and variations in potential glycosylation sites as well as in predicted epitopes. The results of phylogenetic analysis indicated that HA and NA gene segments of H9N2 including those from current and proposed vaccine strains belong to two different Eurasian phylogenetic lineages confirming possible genetic reassortments.

**Conclusions:**

These findings support the continuous evolution of avian H9N2 viruses towards human as host and are in favor of effective surveillance and better characterization studies to address this issue.

## Background

The H9N2 influenza A viruses have been known to cause infection in the poultry population around the globe including Ireland, Iran, Germany, Italy, Pakistan, Saudi Arabia, South Africa and USA since mid-1990 s [1]. In 1998, domestic pigs from Hong Kong were also observed to be infected with H9N2 influenza Y280-like viruses [2]. Several human cases of H9N2 infection have been recorded since 1997 from Hong Kong and China in children and adults exhibiting influenza like symptoms and mild upper respiratory tract infections [2-6]. Genetic analysis of H9N2 viruses from Hong Kong live bird markets showed the preferential binding of viruses to 2, 6-linked sialic acid, human-like receptors [6,7]. All these findings pointed towards the possibility of interspecies transmission of H9N2 viruses and its persistent threat to the human population.

Influenza viruses belonging to the *Orthomyxoviradae *family of viruses are divided into eight single stranded RNA segments encoding ten proteins. These include two surface glycoproteins, hemagglutinin (HA) and neuraminidase (NA), along with nucleoproteins (NP), three polymerase proteins (PA, PB1, PB2) two matrix proteins (M1, M2) and non-structural proteins (NS1, NS2) [8-11]. Of these ten proteins HA and NA are primarily responsible for facilitating influenza virus infection. There are 16 HA and nine NA subtypes. HA is involved in the early stages of infection, causing the binding of the sialic acid receptor present on the host cell surface, and leading to fusion of the viral and endosomal membrane and subsequent entry into the host cell [11]. Virus aggregation is prevented by the NA glycoprotein and by the cleavage of the α-ketosodic linkage between sialic acid and an adjacent sugar residue. This facilitates the movement of the virus to and from the site of infection by destruction of receptors recognized by HA [12]. Previous studies have defined two distinct lineages of H9N2 influenza viruses: North American and Eurasian. The Eurasian lineage can be further divided into three major sublineages; the G1 lineage, represented by A/Quail/Hong Kong/G1/97 (G1-like); the Y280 lineage, represented by three prototype viruses A/duck/Hong Kong/Y280/97 (Y280-like), A/Chicken/Beijing/1/94 (BJ94-like), and A/Chicken/Hong Kong/G9/97 (G9-like) and the Korean lineage, represented by A/chicken/Korea/38349-p96323/96 (Korean-like) and A/duck/Hong Kong/Y439/97 (Y439-like) [7,13,14].

It is important to study the evolution of H9N2 viruses because of their constant prevalence in poultry flocks and repeated emergence in the human population. The present study involved computational molecular analysis and phylogenetic characterization of 11 influenza A (H9N2) viruses which have been isolated between 1997 and 2009. The aim of this study was to aid in understanding the evolution of pandemic H9N2 strains, which have circulated various animal populations in the indicated period.

## Methods

### Viruses

To perform this study, a computational search of all reported cases of influenza A H9N2 human infections from 1997 to 2009 was conducted. A total of eleven nucleotide and their respective deduced amino acid sequences for each of hemagglutinin (HA) and neuraminidase (NA) segments were retrieved from the NCBI flu database [15] accessed on April, 19, 2010. The viruses used in this study are listed in Table 1. Sequencing data was obtained together with information of the host, subtype, isolation year, and isolation place. The selected sequences of H9N2 human cases were then aligned and compared by using multiple sequence alignment software ClustalW2 [16].

**Table 1:** GenBank accession numbers genes and proteins of avian influenza A (H9N2) viruses isolated from 1997 to 2009 from humans.

		Accession Numbers			
		
Year	Strains	Hemagglutinin		Neuraminidase	
		
		Genes	Proteins	Genes	Proteins
1997	A/Hong Kong/1074/97	GU053179.1	ACZ48627	GU053180.1	ACZ45242

1998	A/Shantou/239/98	AY043015.1	AAL32476	AY043021.1	AAL32481
	A/Shaoguan/408/98	AY043017.1	AAL32477	AY043022.1	AAL32482
	A/Shaoguan/447/98	AY043018.1	AAL32478	AY043023.1	AAL32483

1999	A/Hong Kong/1073/99**^a^**	AJ404626.1	CAB95856	AJ404629.1	CAB95859
	A/Hong Kong/1074/99	AJ404627.1	CAB95857	AJ404628.1	CAB95858
	A/Guangzhou/333/99	AY043019.1	AAL32479	AY043024.1	AAL32484

2003	A/Hong Kong/2108/03	DQ226106.1	ABB58945	DQ226128.1	ABB58956

2008	A/Hong Kong/3239/08	CY055156.1	ADC41863	CY055158.1	ADC41865

2009	A/Hong Kong/33982009**^b^**	CY055140.1	ADC41843	CY055142.1	ADC41845
	A/Hong Kong/35820/09	CY055148.1	ADC41853	CY055150.1	ADC41855

**^a ^**A(H9N2) Available vaccine virus (G1 Clade; WHO report, 2010)

**^b ^**A(H9N2) Proposed vaccine virus (G1 Clade; WHO report, 2010)

### Potential Glycosylation sites and antigenic variations

Identification and comparison of N-glycosylation sites into reported protein sequences of HA and NA was performed by an online server ScanProsite [17] and the extent of antigenic variations between viruses was checked by the CTL epitope prediction method [18]. Each amino acid sequence of HA and NA was evaluated separately using consensus approach.

### Phylogenetic characterization and tree construction

Phylogenetic patterns of NA and HA nucleotide sequences of H9N2 influenza viruses isolated between 1997 and 2009 from humans were observed using MEGA4.0.2 [19]. The selected nucleotide sequences based on local alignment and homology searches using BLAST were aligned by using CLUSTALW. Unrooted phylogenetic trees were constructed by using minimum evolution analysis with maximum composite likelihood and the Tamura-Nei model. Internal branching probabilities were determined by bootstrap analysis of 1000 replicates and are indicated by percentage value on each branch.

## Results and Discussion

### Molecular Analysis

Influenza A viruses of subtype H9N2 are now considered to be widespread in poultry and have demonstrated the ability to infect humans [20]. The recurring presence of H9N2 infections in humans has raised concerns about the possibility of H9N2 viruses evolving into pandemic strains. Therefore, it is crucial to evaluate the potential pandemic threat posed by H9N2 viruses using experimental and computational approaches. During this study, bioinformatics analysis of HA and NA from H9N2 viruses was performed and the key residues in receptor binding sites (RBS), the cleavage motifs of HA and NA hemadsorbing sites (HB), stalk length and enzyme active sites were studied in detail.

#### Hemagglutinin

It has been well documented that the receptor binding site motif of HA is critical for cellular receptor specificity and determining virus host range [21,22]. Out of five conserved amino acids in the pocket of the HA glycoprotein, two positions showed the maximum number of mutations (198, 234) and three remained 99% conserved (191, 235, and 236). The pattern of observed mutations at position 198 was E^198^T, E^198^A, E^198^V and E^198^D, whereas for position 234 was L^234^Q, Q^234^L and Q^234^M as summarized in Table 2. Mutations in these regions are considered strong factors for change in sugar specificity thus leading to change in host specificity. The presence of glutamine (Q) at position 234 (H3 numbering: 226) is a typical avian virus signature, and it has been reported that presence of this amino acid results in a preference for binding to 2,3-linked sialic acid (avian receptors) whereas, in the case of leucine (L) at the same position, there is a preference for 2,6-linked sialic acid (human receptors) and potential cause of reported human infections [5,6,23-25]. However, upon analysis of amino acids at the receptor binding site of HA glycoproteins, we have identified that H9N2 viruses isolated in 2009 from infected patients in Hong Kong possessed glutamine at position 234 (H3 numbering: 226) instead of leucine as has been found in previous isolates, yet still somehow managed to bind with human sialic receptors. As shown in Table 2, this residue variation in avian H9N2 viruses has not been reported before in human cases. This observation can be correlated with a number of phenomena such as genetic evolution of influenza viruses in order to evade host defense mechanisms, and transfer of this virus from swine to humans instead of avian to human transmission. It is also known that pigs act as a "mixing vessel" because viruses isolated from pigs recognize both types of sialic receptors [26] and most importantly represent the balance that exists between the activities of HA and NA. Both the genes PB2 and HA are known to be critical for the pathogenicity of the virus. Therefore, the role of other proteins such as the PB2 segment [4,27] must be viewed as potential causes of zoonotic H9N2 possibly resulting in future human to human transmission.

**Table 2:** Comparison of critical amino acid residues in hemagglutinin and neuraminidase proteins.

	Hemagglutinin						Neuraminidase																										
	
Viruses	Receptor Binding Site					cleavage Site	Stalk deletions			Hemadsorbing Site																	drug binding pocket						
	**191**	**198**	**234**	**235**	**236**	**335 - 338**	**38-39**	**46-50**	**62-64**	**366 - 373**								**399 - 404**						**431 - 433**			**118**	**119**	**151**	**152**	**277**	**292**	**371**

	**H**	**E**	**L**	**Q**	**G**	**RSSR**				**I**	**K**	**K**	**D**	**S**	**R**	**S**	**G**	**D**	**S**	**D**	**I**	**R**	**S**	**P**	**Q**	**E**	**R**	**E**	**D**	**R**	**E**	**R**	**R**

A/Quail/Hong Kong/G1/97^1^	-	-	-	-	-	-	Yes	No	No	-	-	-	-	-	-	-	-	-	-	-	-	-	-	-	-	-	-	-	-	-	-	-	-

A/Duck/Hong Kong/Y280/97^2^	-	T	-	-	-	-	No	No	Yes	-	-	E	-	-	-	-	-	-	-	-	N	W	-	-	-	-	-	-	-	-	-	-	-

A/Chicken/Beijing/1/94^2^	-	V	Q	-	-	-																											

A/Chicken/Hong Kong/G9/97^2^	-	A	-	-	-	-																											

A/Duck/Hong Kong/Y439/97^3^	-	-	Q	-	-	A-N-																											

A/Ck/Korea/38349-p96323/96^3^	-	-	Q	-	-	A-Y-	No	No	No	-	S	-	-	-	-	-	-	-	N	N	N	W	-	-	-	-	-	-	-	-	-	-	-

A/Hong Kong/1074/97	-	-	-	-	-	-	Yes	No	No	-	-	-	-	-	-	A	-	-	-	-	N	W	-	-	-	-	-	-	-	-	-	-	-

A/Shantou/239/98	N	A	-	-	-	-	No	No	No	-	-	-	-	-	-	-	-	-	-	-	S	S	-	-	K	-	-	-	-	-	-	-	-

A/Shaoguan/408/98	N	A	-	-	-	-	No	No	Yes	-	-	E	-	-	-	-	-	-	-	-	N	W	-	-	-	-	-	-	-	-	-	-	-

A/Shaoguan/447/98	N	A	-	-	-	-	No	No	Yes	-	-	E	-	-	-	-	-	-	-	-	N	W	-	-	-	-	-	-	-	-	-	-	-

A/Hong Kong/1073/99^a^	-	-	-	-	-	-	Yes	No	No	-	-	-	-	-	-	-	-	-	-	-	N	W	-	-	-	-	-	-	-	-	-	-	-

A/Hong Kong/1074/99	-	-	-	-	-	-	Yes	No	No	-	-	-	-	-	-	-	-	-	-	-	N	W	-	-	-	-	-	-	-	-	-	-	-

A/Guangzhou/333/99	N	V	M	-	-	-	No	No	No	-	E	-	-	-	-	-	-	-	-	-	N	S	-	-	K	-	-	-	-	-	-	-	-

A/Hong Kong/2108/03	N	V	-	-	-	-	No	No	No	-	E	-	-	-	-	-	-	-	R	-	N	S	-	-	K	-	-	-	-	-	-	-	-

A/Hong Kong/3239/08	N	A	-	-	-	-	No	No	No	-	E	-	-	-	-	-	-	-	-	-	N	S	-	-	K	-	-	-	-	-	-	-	-

A/Hong Kong/33982/09^b^	-	D	Q	-	-	--N-	No	No	No	-	E	-	-	-	-	-	-	-	R	-	N	S	-	-	K	-	-	-	-	-	-	-	-

A/Hong Kong/35820/09	-	D	Q	-	-	--N-	No	No	No	-	E	-	-	-	-	-	-	-	R	-	N	S	-	-	K	-	-	-	-	-	-	-	-

Amino acid residues were compared in the hemagglutinin receptor binding site and cleavage sites, as well as in the neuraminidase stalk region, HB site and antiviral drug binding sites of influenza A H9N2 viruses isolated during 1997-2009 from humans. Residues differences and similarities are compared with reference strains of lineages (G1-like^1^, Y280-like^2 ^and Korean-like^3^) and vaccine strains

^a, b^. Dashes indicate identical/conserved residues.

**^a ^**A(H9N2) Available vaccine virus (G1 Clade; WHO report, 2010) **^b ^**A(H9N2) Proposed vaccine virus (G1 Clade; WHO report, 2010)

Analysis of the HA cleavage site showed that strains isolated in 2009 have a different cleavage site compare to those previously reported. From 1997 to 2008, all H9N2 viruses retained a conserved amino acid pattern at the cleavage site; ^335^RSSR^338^GLF^341 ^except for the sublineage prototype strain (A/chicken/Kr/38349-p96323/96) that had the cleavage site ^335^ASYR^338^GLF^341^. Presence of the R-S-S-R motif is suggestive of H9N2 viruses with low pathogenicity that have adapted to chicken host [28-30]. However, it has been observed that viruses isolated in 2009 have the different pattern (^335^RSNR^338^GLF^341^) due to the substitution mutation S^337^N (Table 2). To our knowledge, this is the first report to show presence of the R-S-N-R motif in avian H9N2 influenza viruses isolated from human patients although the R-S-N-R motif had been reported before in different studies on avian H9N2 viruses from around the globe [31-33]. The significance of this mutation on viral stability or increased pathogenicity is not fully understood as yet and requires further studies, as the cleavage site is considered an indicator of pathogenicity [34,35]. Comparison with vaccines strains also revealed variations at positions 198 and 234 in the HA glycoprotein. The currently available vaccine virus to the virus has an E and an L at positions 198 and 234, respectively, whereas strains associated with human infections reported in 2009 contain D and Q residues at these respective positions.

#### Neuraminidase

The major factors known to influence the functional activities of the NA glycoprotein are enzyme active sites, the stalk length, HB site and potential glycosylation sites. The HB site is located on the surface of the NA molecule, away from the neuraminidase enzyme active site [36]. Analysis of the HB site showed substitutions similar to those detected in the Y280 lineage prototype strain (A/Duck/Hong Kong/Y280/97) and in H9N2 human isolates from Hong Kong in 1999. These mutations are typical of human pandemic H2N2 and H3N2 viruses [7,14]. The most prominent mutation is at position 402 from isoleucine to asparagine/serine and at position 403 from arginine to tryptophan/serine. Strains from 1999 - 2009 also showed mutations at position 367 from lysine to glutamic acid and at position 432 from glutamine to lysine as described in Table 2. However, the biological significance of any of these substitutions in the HB site is not yet known.

The NA protein consists of a box-like head with an enzymatic active site that is connected to a fibrous stalk region of variable length. The NA stalk is important for balancing the complementary activity of HA and NA and has been correlated with efficiency of virus replication and pathogenesis. From various studies previously performed it has been concluded that longer stalk length of the virus results in better replication [37-40]. Analysis of stalk length revealed that the two prototype viruses, Qa/Hong Kong/G1/97 and A/Hong Kong/1073/97, of the G1-lineage contained a two amino acid deletion in the NA stalk region at positions 38 and 39, a deletion at position 62 - 64 was observed in A/Duck/Hong Kong/Y280/97, A/Shaoguan/408/98 and A/Shaoguan/447/98. The particular 46-50 amino acid deletion, which is important for poultry adaptation of the virus [41], is not found in any of the analyzed sequences even though these viruses are thought to be transmitted from avian species to humans. Furthermore, as shown in Table 2, from 1999 - 2009 none of the reported strains contained a stalk deletion at any position, which may also be an indication of evolution in the viral structure leading to a NA protein with better replication rate in humans.

Sequence analysis of binding pocket residues in NA for drugs such as zanamivir (Relenza^®^) and oseltamivir (Tamiflu^®^) was also performed. These analyses showed that for each virus, amino acids in the enzyme active site were conserved and no substitution mutations were seen, which may lead to an evolution of sialidase inhibitor resistant viruses (Table 2). It is known that mutations at positions other than the active sites still tend to alter the activity of active site amino acids; therefore this possibility cannot be excluded. Such mutations are not always captured or extensively studied in homology models. Therefore, wet lab studies are required to check the level of effectiveness possessed by NA inhibitors against recently reported strains.

### Phylogenetic characterization

Evolutionary relationships of HA and NA nucleotide sequences were determined by comparing H9N2 human isolates from 1997 - 2009 with the established Eurasian H9N2 lineages: namely, the G1, Y280 and Korean-lineages represented by their respective prototype viruses (Table 1). The strains of current and proposed vaccines were also included in this analysis.

**Figure 1:**
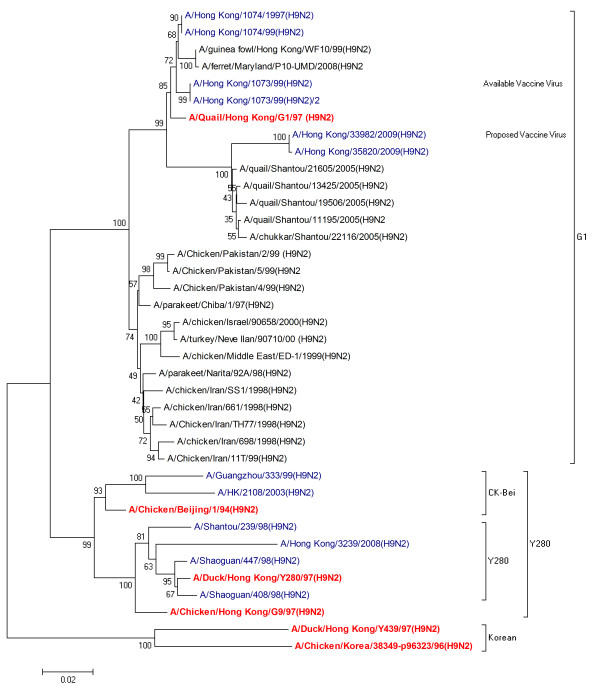
**Phylogenetic relationships of HA genes in H9N2 influenza viruses isolated from humans between 1997 and 2009**. A phylogenetic tree was generated using minimum evolution analysis with maximum composite likelihood using the Tamura-Nei model with MEGA software version 4.0.2. Numbers below branches indicate bootstrap value percentages from 1000 replicates. The scale bar represents the distance unit between sequence pairs. Representative prototype viruses for different Eurasian lineages are indicated as red. The sequences of H9N2 influenza viruses isolated from the human population are indicated as blue.

Two distinct groups were observed in the unrooted phylogenetic tree for HA (Figure 1). Viruses isolated from Hong Kong in 1997, 1999, and in 2009 showed a more intimate relationship with the G1 lineage strain and clustered together in one group. Sequences from Hong Kong isolated in 2003 and 2008, along with Chinese strains from 1998 and 1999 showed the close association with the Y280 lineage and clustered together in second group. None of the reported sequences clustered within the Korean-like lineage.

**Figure 2:**
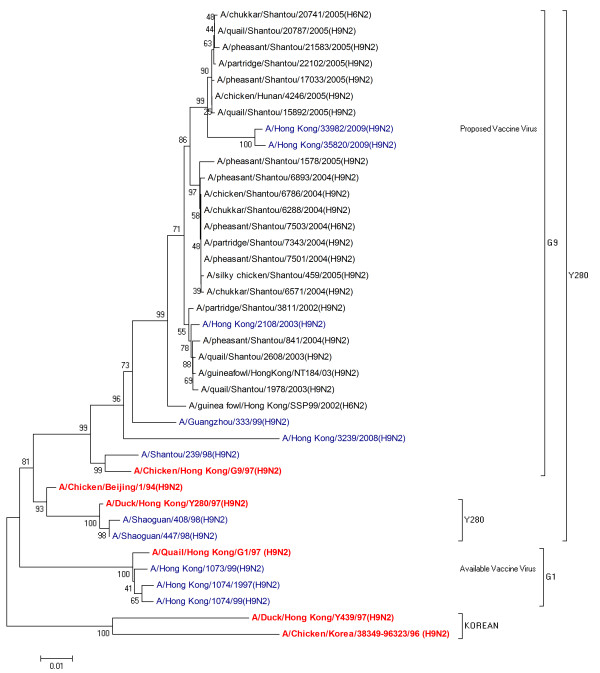
**Phylogenetic relationships of NA genes in H9N2 influenza viruses isolated from humans between 1997 and 2009**. The phylogenetic methods and abbreviations were as described for figure 1.

The phylogenetic tree for NA (Figure 2) showed clustering pattern different from that observed in the HA phylogenetic results. Similar to HA, none of the sequences grouped within the Korean-like lineage. However, sequences isolated from Hong Kong in 2003, 2008 and 2009 clustered within G9-like lineage which is one of the prototype strains from the Y280 lineage. It was observed that the proposed vaccine strain and the 2009 strain from Hong Kong appear in the same sub group with 100% similarity. Sequences isolated from Shaoguan, China in 1998 clustered together with the Y280 lineage strain in one subgroup, while another contained the G1 lineage strain along with available vaccine strain and strains from Hong Kong isolated in 1997 and 1999.

Collectively, we observed that phylogenetic relationship of strains at different times and geographical regions displayed complexity and diversity. It was identified that both HA and NA glycoproteins do not belong to a single lineage but originate from different Eurasian sublineages which relates to genetic heterogeneity of H9N2 viruses. These results were also in consensus with the sequence analysis results of strains exhibiting high sequence similarity in the same lineages and sublineages.

### N-Glycosylation sites of HA and NA

The N-linked glycosidic moieties have been found to play a vital role in mediating viral infectivity, receptor binding capacity and cell associated host immune responses, as well as protecting critical viral protein epitopes from immune attack [42,43]. Potential glycosylation sites with the N-X-T/S motif (in which X may be any amino acid except proline) were identified, which revealed the region based similarities and yearly variations. For H9N2 HA protein sequences, the N-glycosylation sites of viruses isolated from Hong Kong in 1997 were similar to those isolated in 1999 (current vaccine strain) from Hong Kong. The strains isolated from China in 1998 exhibited a different pattern of glycosylation sites in terms of position and sequence pattern. A similar case was observed for another virus isolated from China (A/Guangzhou/333/99) in 1999, which showed a totally different pattern of glycosylation sites as shown in Table 3. It may be because viruses from China fall into the Y280 lineage while other cases are closest to the G1 sublineage as shown in our phylogenetic analysis. Sequence analysis results also showed that Chinese isolates contained mutations in the HA receptor binding site at positions 191N and 198A, as compared to other viruses having 191 H and 198E or 198 D (Table 2). Viruses from Hong Kong isolated during 2003 contained altogether different N- glycosylation sites at different positions, which were not found in the analysis of previous entries. Glycosylation sites of viral strains from 2008 and 2009 again had high similarity with each other but were different from all the previous strains except at one site with a single mutation 305 - 308 (NISK - NCSK) in 2008 strains, which was also present in Hong Kong viruses from 1997 - 1999.

**Table 3:** Comparison of predicted N-glycosylation sites of the virus hemagglutinin proteins.

Hemagglutinin (HA)													
**1997**		**1998**		**1999**				**2003**		**2008**		**2009**	

**A/Hong Kong/1074/97**		**A/Shantou/239/98** **A/Shaoguan/408/98** **A/Shaoguan/447/98**		**A/Hong Kong/1073/99** **A/Hong Kong/1074/99**		**A/Guangzhou/333/99**		**A/Hong Kong/2108/03**		**A/Hong Kong/3239/08**		**A/Hong Kong/33982/09** **A/Hong Kong/35820/09**	

**Position**	**Sequence**	**Position**	**Sequence**	**Position**	**Sequence**	**Position**	**Sequence**	**Position**	**Sequence**	**Position**	**Sequence**	**Position**	**Sequence**

29 - 32	NSTE	12 - 15	NSTE	29 - 32	NSTE	124 - 127	NVTY	19 - 22	NSTE	21 - 24	NSTE	21 - 24	NSTE

105 - 108	NGTC	124 - 127	NVSY	105 - 108	NGTC	281 - 284	NTTL	131 - 134	NVTY	133 - 136	NVSY	97 - 100	NGTC

141 - 144	NVTY	201 - 204	NRTF	141 - 144	NVTY	288 - 291	NVSK	288 - 291	NTTL	210 - 213	NRTF	133 - 136	NVTY

206 - 209	NDTT	281 - 284	NTTL	206 - 209	NDTT	475 - 478	NGTY	295 - 298	NVSK	290 - 293	NTTL	198 - 201	NDTT

218 - 221	NRTF	288 - 291	NVSK	218 - 221	NRTF			482 - 485	NGTY	297 - 300	NVSK	290 - 293	NSTL

298 - 301	NSTL	475 - 478	NGTY	298 - 301	NSTL					305 - 308	NCSK	297 - 300	NISK

305 - 308	NISK			305 - 308	NISK					484 - 487	NGTY	484 - 487	NGTY

492 - 495	NGTY			492 - 495	NGTY								

551 - 554	NGSC			551 - 554	NGSC								

Similar to HA, NA sequences isolated from Hong Kong in 1997 and 1999 showed similar glycosylation sites, as did the sequences isolated from China in 1999 and from Hong Kong in 2003. Sequences isolated from Hong Kong in 2008 and 2009 also had similar glycosylation sites. An interesting observation was that in some cases of HA and NA, the glycosylation sites remained the same as those seen in previous or later strains. However, there was a change of position for these sites, such as 198 - 201 (NATA) in Hong Kong/1074/1997 and 183 - 186 (NATA) in Shantou/239/98 (Table 4).

**Table 4:** Comparison of predicted N-glycosylation sites of the virus neuraminidase proteins.

1997		1998						1999				2003		2008		2009	
**A/Hong Kong/1074/97**		**A/Shantou/239/98**		**A/Shaoguan/408/98**		**A/Shaoguan/447/98**		**A/Hong Kong/1073/99** **A/Hong Kong/1074/99**		**A/Guangzhou/333/99**		**A/Hong Kong/2108/03**		**A/Hong Kong/3239/08**		**A/Hong Kong/33982/2009** **A/Hong Kong/35820/2009**	

**Position**	**Sequence**	**Position**	**Sequence**	**Position**	**Sequence**	**Position**	**Sequence**	**Position**	**Sequence**	**Position**	**Sequence**	**Position**	**Sequence**	**Position**	**Sequence**	**Position**	**Sequence**

59 - 62	NITE	27 - 30	NSSN	66 - 69	NSTT	47 - 50	NSTT	59 - 62	NITE	44 - 47	NSSN	44 - 47	NSSD	36 - 39	NSSD	36 - 39	NSSD

67 - 70	NNTT	44 - 47	NITE	83 - 86	NWSK	64 - 67	NWSK	67 - 70	NNTT	61 - 64	NITE	61 - 64	NITE	53 - 56	NITE	53 - 56	NITE

68 - 71	NTTI	52 - 55	NSTT	143 - 146	NGTT	124 - 127	NGTT	68 - 71	NTTI	69 - 72	NSTT	69 - 72	NSTT	61 - 64	NSTT	61 - 64	NSTT

84 - 87	NWSK	69 - 72	NWSK	197 - 200	NATA	178 - 181	NATA	84 - 87	NWSK	86 - 89	NWSK	86 - 89	NWSK	138 - 141	NGTS	78 - 81	NWSK

144 - 147	NGTI	129 - 132	NGTS	231 - 234	NGTC	212 - 215	NGTC	144 - 147	NGTI	146 - 149	NGTA	146 - 149	NGTA	192 - 195	NATA	138 - 141	NGTT

198 - 201	NATA	183 - 186	NATA	399 - 402	NWSG	380 - 383	NWSG	198 - 201	NATA	200 - 203	NATA	200 - 203	NATA	226 - 229	NGTC	192 - 195	NATA

232 - 235	NGTC	217 - 220	NGTC					232 - 235	NGTC	234 - 237	NGTC	234 - 237	NGTC	394 - 397	NSSG	226 - 229	NGTC

400 - 403	NWSG							400 - 403	NWSG	402 - 405	NSSG	402 - 405	NSSG			394 - 397	NSSG

### Antigenic sites of HA and NA

Analysis of antigenic sites of HA and NA also showed region based similarities and yearly variations as observed for glycosylation sites distribution. Viruses isolated from Hong Kong in 1997 and 1999 had the same antigenic sites as strains isolated from China in 1998 and 1999, except for A/Shaoguan/447/98 which had only one antigenic site (219 - GRIDYYWSV) similar to other Chinese strains. Strains isolated from Hong Kong in 2003, 2008 and 2009 had similar antigens as the earlier strains from the region but at different positions compared to the strains from 2008 and 2009, but again presenting similar antigenic site positions (Table 5).

**Table 5:** Comparison of antigenic sites in amino acid sequences of hemagglutinin proteins (H9N2).

1997		1998						1999				2003		2008		2009	
**A/Hong Kong/1074/97**		**A/Shantou/239/98**		**A/Shaoguan/408/98**		**A/Shaoguan/447/98**		**A/Hong Kong/1073/99** **A/Hong Kong/1074/99**		**A/Guangzhou/333/99**		**A/Hong Kong/2108/03**		**A/Hong Kong/3239/08**		**A/Hong Kong/33982/2009** **A/Hong Kong/35820/2009**	

**Position**	**Sequence**	**Position**	**Sequence**	**Position**	**Sequence**	**Position**	**Sequence**	**Position**	**Sequence**	**Position**	**Sequence**	**Position**	**Sequence**	**Position**	**Sequence**	**Position**	**Sequence**

431	AYNAELLVL	414	AYNAELLVL	414	AYNAELLVL	253	GESHGRILK	431	AYNAELLVL	414	AYNAELLVL	421	AYNAELLVL	423	AYNAELLVL	423	AYNAELLVL

108	CYPGNVENL	91	CYPGNVENL	91	CYPGNVENL	473	IRNGTYNRR	108	CYPGNVENL	91	CYPGNVENL	98	CYPGNVENL	100	CYPGNVENL	100	CYPGNVENL

236	GRIDYYWSV	219	GRIDYYWSV	428	TLDDHDANV	219	GRIDYYWSV	236	GRIDYYWSV	219	GRIDYYWSV	226	GRIDYYWSV	228	GRIDYYWSV	228	GRIDYYWSV

312	GTCPKYVRV	117	IFPDTIWNV	219	GRIDYYWSV	360	DSTQKAIDK	312	GTCPKYVRV	82	ERPSAVNEM	124	IFPDTIWNV	126	IFPDTIWNV	304	GTCPKYVKV

134	IFPDTTWNV	392	SEVENRLNM	117	IFPDTIWNV	396	TRLNMINNK	134	IFPDTTWNV	117	IFPDTIWNV	314	KLAVGMRNV	316	KLAVGLRNV	316	KLAIGLRNV

For NA, the sequences isolated from Hong Kong in 1997 and 1999 had the same antigenic sites. Sequences from China isolated in 1998 had similar antigenic sites but at different positions with variation at one site. Chinese sequences from 1999 had three sites that were the same as those in the Hong Kong viruses isolated during 2003. Sequences isolated from Hong Kong in 2008 and 2009 also had similar antigenic sites as previous strains from the region but at different positions. Furthermore, in 2008, one new antigenic site was observed in a sequence (102 - ASGDIWVTR) and another two in 2009 sequences at positions 19 (ALFATTMTL) and 240 (GRADTRILF) as shown in Table 6. Another observation was that both the available and proposed vaccine strains are not fully coordinated with the antigenic epitope regions of the HA and NA genes. Therefore, in case of reappearance of incompatible viral strains, vaccine products may become less effective.

**Table 6:** Comparison of antigenic sites in amino acid sequences of neuraminidase proteins (H9N2).

1997		1998						1999				2003		2008		2009	
**A/Hong Kong/1074/97**		**A/Shantou/239/98**		**A/Shaoguan/408/98**		**A/Shaoguan/447/98**		**A/Hong Kong/1073/99** **A/Hong Kong/1074/99**		**A/Guangzhou/333/99**		**A/Hong Kong/2108/03**		**A/Hong Kong/3239/08**		**A/Hong Kong/33982/2009** **A/Hong Kong/35820/2009**	

**Position**	**Sequence**	**Position**	**Sequence**	**Position**	**Sequence**	**Position**	**Sequence**	**Position**	**Sequence**	**Position**	**Sequence**	**Position**	**Sequence**	**Position**	**Sequence**	**Position**	**Sequence**

98	FSKDNSIRL	83	FSKDNSIRL	97	FSKDNSIRL	78	FSKDNSIRL	98	FSKDNSIRL	100	FSKDNSIRL	100	FSKDNSIRL	92	FSKDNSIRL	92	FSKDNSIRL

295	GSNRPVLYI	280	GSNRPVLYI	294	GSNRPVLYI	275	GSNRPVLYI	295	GSNRPVLYI	248	GRADTRILF	297	GSNRPVLYI	289	GSNRPVLYI	19	ALFATTMTL

459	GANINFMSI	231	GRADTRILF	152	HRTLLMNEL	133	HRTLLMNEL	459	GANINFMSI	155	HRTLLMNEL	248	GRADTRILF	102	ASGDIWVTR	240	GRADTRILF

153	HRTLLMNEL	138	HRTLLMNEL	145	TTHDRIPHR	126	TTHDRIPHR	153	HRTLLMNEL	378	RVIGGWITA	155	HRTLLMNEL	147	HRTLLMNEL	289	GSNRPVLFI

277	SCYPRYPEV	282	NRPVLYINM	296	NRPVLYINM	277	NRPVLYINM	277	SCYPRYPEV	279	SCYPRYPEV	458	WPDGANINL	291	NRPVLYINM	102	AAGDIWVTR

The potential for differences in antigenic variations is high, especially when compared across sublineages of H9N2 viruses. Xu et al [44] demonstrated antigenic diversity in H9N2 viruses using monoclonal antibodies, which corresponded with phylogenetic relationships. Findings of the study by Xu et al also correspond with our results where H9N2 viruses showed sequence variations and antigenic diversity based on evolution. Furthermore, differences in glycosylation of the virion surface proteins may contribute to antigenic variations, however this requires further evaluation.

## Conclusions

In summary, we have reported an *in silico *molecular analysis of HA and NA genes and respective deduced amino acid sequences from H9N2 avian influenza viruses that were isolated from humans between 1997 and 2009. We found that H9N2 surface genes belonged to two distinct lineages - G1 and Y280, indicating that they have different sources of origin. Sequence analysis revealed unique variations in antigenic and N - linked glycosylation sites. Drug binding pockets remained highly conserved in all reported strains and hence, the activity of NA inhibitors should remain unaffected. However, matching with vaccine strains showed variations, requiring further investigations in animal models. The RBS modification of leucine to glutamine (Leu^226^Glu) instead of glutamine to leucine and a new cleavage site motif (R-S-N-R) for HA is related to the balancing activity of NA. Findings from the study support the genetic instability of influenza A (H9N2) viruses and highlight the necessity for more comprehensive surveillance and further evaluation of H9N2 viruses with proper *in vitro *and *in vivo *models.

## Abbreviations

HA: Hemagglutinin; NA: Neuraminidase; PGS: Potential Glycosylation Sites; RBS: Receptor Binding Site; HB: Hemadsorbing Site.

## Competing interests

The authors declare that they have no competing interests.

## Authors' contributions

AMB designed the study, performed molecular analysis and drafted the manuscript. SS performed phylogenetic and antigenic analysis. MI and YT analyzed the data. YT finalized the manuscript. All authors have read and approved the final manuscript.
